# Assessment of candidate elements for development of spectral photon-counting CT specific contrast agents

**DOI:** 10.1038/s41598-018-30570-y

**Published:** 2018-08-14

**Authors:** Johoon Kim, Daniel Bar-Ness, Salim Si-Mohamed, Philippe Coulon, Ira Blevis, Philippe Douek, David P. Cormode

**Affiliations:** 1 Department of Radiology, University of Pennsylvania, 3400 Spruce St, 1 Silverstein, Philadelphia, PA 19104 USA; 2 Department of Bioengineering, University of Pennsylvania, 3400 Spruce St, 1 Silverstein, Philadelphia, PA 19104 USA; 3 Department of Medicine, Hôpital Cardio-Vasculaire et Pneumologique Louis Pradel, Lyon, France; 4 Centre de Recherche en Acquisition et Traitement de l’Image pour la Santé (CREATIS), UMR CNRS 5220, Inserm U1044, University Lyon1 Claude Bernard, Lyon, France; 5 CT Clinical Science, Philips, Suresnes, France; 6 Global Advanced Technologies, CT, Philips, Haifa, Israel

## Abstract

Spectral photon-counting computed tomography (SPCCT) is a rapidly emerging imaging modality that provides energy-dependent information on individual x-ray photons, leading to accurate material decomposition and simultaneous quantification of multiple contrast generating materials. Development of SPCCT-specific contrast agents is needed to overcome the issues with currently used iodinated contrast agents, such as difficulty in differentiation from calcified structures, and yield SPCCT’s full promise. In this study, the contrast generation of different elements is investigated using a prototype SPCCT scanner based on a modified clinical CT system and suitable elements for novel contrast agent development for SPCCT imaging are identified. Furthermore, nanoparticles were synthesized from tantalum as a proof of concept spectral photon-counting CT agent and tested for their *in vitro* cytotoxicity and contrast generation to provide insight into the feasibility of nanoparticle contrast agent development from these elements. We found that gadolinium, ytterbium and tantalum generate high contrast in spectral photon-counting CT imaging and may be suitable elements for contrast agent development for this modality. Our proof of concept results with tantalum-based nanoparticles underscore this conclusion due to their detectability with spectral photon-counting CT, as well as their biocompatibility.

## Introduction

X-ray computed tomography (CT) is one of the most widely used imaging modalities in medicine due to its broad availability, low cost, high spatial resolution and fast image acquisition^1^. Spectral photon-counting CT (SPCCT) is a rapidly emerging form of CT that uses a standard polychromatic x-ray source and photon-counting detectors (PCDs)^2–9^. Prototypes of clinical-grade SPCCT scanners have been modified from clinical CT systems and are currently available for both pre-clinical and clinical research^4,6,7^. The energy integrating detectors (EIDs) used in conventional CT scanners discard the energy information of incident x-ray photons, since they sum all the energy deposited in each pixel, resulting in photons with higher energy being weighted more heavily than photons with lower energy^10,11^. PCDs, on the other hand, measure the energy of individual x-ray photons by converting x-rays into currents, inferring their energies via pulse height analysis, and separating them into several data bins with adjustable energy thresholds^12^. Since different materials have differing x-ray attenuation profiles, characterizing the energy distribution of the transmitted beam by SPCCT systems allows the specific detection of substances such as exogenous contrast agents^6,13^.

SPCCT systems have multiple benefits, such as improved contrast-to-noise ratios (CNR), lower noise, higher spatial resolution and fewer image artifacts, such as blooming and beam hardening^3,11,14,15^. These benefits can lead to the acquisition of higher quality images at lower radiation doses than with conventional CT systems. Preclinical and clinical studies have demonstrated the advantages of SPCCT imaging with several animal models, as well as for patients^2,4,7,16,17^.

Besides the advantages of SPCCT in terms of image quality and patient dose, these systems can specifically distinguish multiple contrast generating materials in a single scan via material decomposition by assigning an appropriate number of energy bins and their thresholds^18–23^. This feature allows differentiation of exogenous contrast agents from soft tissues and calcified structures (e.g. bones and calcified atherosclerotic plaques), which can be especially beneficial in coronary CT angiography. Simultaneous material decomposition also eliminates the need for comparison of pre- and post-injection images, which can further reduce patient radiation exposure. As demonstrated by Si-Mohamed *et al*.^6^, SPCCT systems are also capable of providing absolute quantification of exogenous contrast agents *in vivo* to allow biodistribution determination without the need for *ex vivo* analysis.

**Figure 1:**
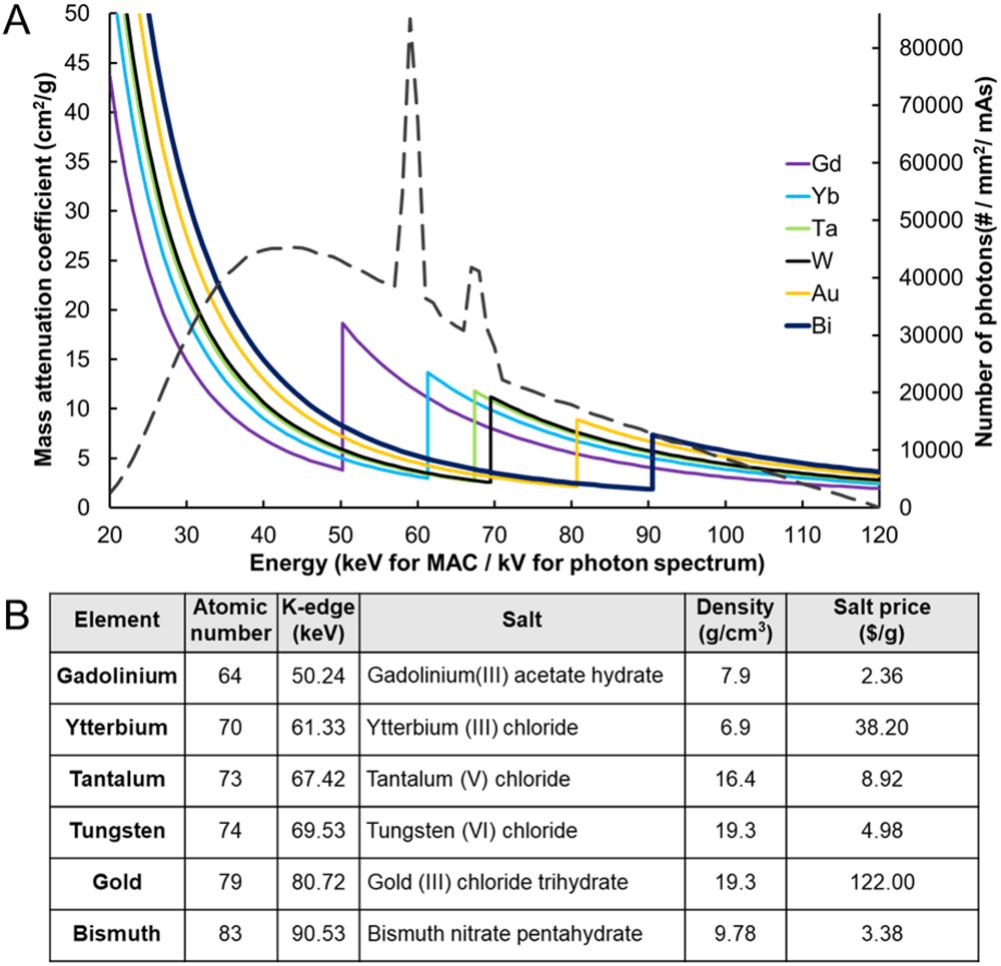
(**A**) Mass attenuation coefficients of various heavy metal elements and x-ray photon intensities at a tube voltage of 120 kV. The x-ray photon intensity spectrum (dotted line) was generated by using Spektr 3.0 (x-ray spectrum modeling software). (**B**) Characteristics of the six heavy elements used in this study. The salt prices of these elements were obtained from Sigma-Aldrich.

Iodine-based small molecules are the most commonly used intravenous contrast agents for CT imaging. While contrast generation of iodinated contrast agents in SPCCT systems is as effective as contrast generation in conventional and dual energy CT systems, they do not take full advantage of the capability of K-edge imaging in SPCCT since iodine’s K-edge energy (33.2 keV) is too low for material decomposition for most clinical CT applications. This is because there are too few photons in CT x-ray beams below this K-edge for accurate data to be gathered. Iodine-based contrast agents are also known to cause allergic reactions, and there are concerns over kidney function reduction in patients with renal insufficiency through contrast-induced nephropathy (CIN) or contrast-induced acute kidney injury^14,24,25^. CIN does not develop into chronic renal failure in most cases; however, it may still result in complete renal failure in the patients with poor renal functions, increasing the risk of morbidity and mortality^14,26^. Thus, developing novel contrast agents specifically designed for SPCCT can be valuable to allow full utilization of its capabilities and broaden its applications. Substantial work has been done over the past decade using nanoparticles based on elements such as gold^27–31^, bismuth^32,33^, tantalum^34,35^ and others^36–40^ as contrast agents for EID-based CT. These nanoparticle-based CT contrast agents can be synthesized to have similar or different pharmacokinetics and biodistributions to iodine. They have been studied for various applications, such as vascular imaging^29,41^, theranostics in drug delivery^42^ and radiotherapy^43^, as well as cell tracking^44,45^. Contrast agents for SPCCT imaging need to be based on elements that have K-edge energies within a region where there are a reasonable number of photons above and below their K-edge (roughly 40–100 keV in a 120 kVp beam) for effective imaging (Fig. 1A). There have been a number of reports focusing on the use of contrast agents for SPCCT that are based on elements such as gold, bismuth or gadolinium^2,5,13,18,21^. However, to the best of our knowledge, a systematic study of the contrast generation from different elements in SPCCT imaging has not been reported to date.

We therefore decided to survey the elements for candidates for novel SPCCT contrast agents. From elements whose K-edge fell into the range of 40–100 keV, we first eliminated those that are highly toxic or radioactive, such as thulium and radium^14^. Then we considered economically viable elements that have been previously studied as experimental contrast agents or for nanoparticle syntheses. Taking those criteria into consideration, we selected gadolinium, ytterbium, tantalum, tungsten, gold and bismuth as the most suitable elements for contrast agent development (Fig. 1B). Gold is the element that has been most commonly used as a contrast generating material for SPCCT^6,18,46^, therefore we included it in this panel despite its relatively high material cost. We herein report the evaluation of the contrast production of these elements using a prototype SPCCT system with a high count rate high enough to cope with photon fluxes needed for medical imaging. Nanoparticle-based contrast agents from a lead element can allow successful material differentiation from calcified structures to broaden the applications of SPCCT imaging^47^. They can also be synthesized to have favorable pharmacokinetics and biodistribution, as well as to act as a platform for targeted imaging. Therefore, we formed nanoparticles with a lead element and performed proof of concept experiments with this material that demonstrated its potential as an SPCCT agent to provide insight into the feasibility of contrast agent development from these elements. By identifying elements that produce high CNR in a prototype SPCCT scanner, this study provides guidance for the development of novel SPCCT-specific contrast agents.

## Methods

### Materials

Gadolinium(III) acetate hydrate (99.9% trace metals basis), ytterbium(III) chloride hexahydrate (99.9%), tantalum(V) chloride (99.8%), tungsten(VI) chloride (99.9%), gold(III) chloride trihydrate (99.9%), bismuth(III) nitrate pentahydrate (98.0%), tantalum(V) ethoxide (99.98%), cyclohexane (99%), IGEPAL CO-520, ammonium hydroxide solution (28.0–30.0% NH_3_ basis) and calcium phosphate (96.0%) were purchased from Sigma-Aldrich (St. Louis, MO). 2-(carbomethoxy)ethyltrimethoxysilane and 3-(trimethoxysilyl)propyl-N,N,N-trimethylammonium chloride were obtained from Gelest, Inc (Morrisville, PA). Iomeron 400 mg/ml was purchased from Bracco (Milan, Italy). The cell lines used for *in vitro* experiments, HepG2, Renca, SVEC4-10EHR1, were provided by ATCC (Manassas, VA). LIVE/DEAD assay kits were purchased from Life Technologies Invitrogen (Grand Island, NY).

### SPCCT and conventional CT systems

**Figure 2:**
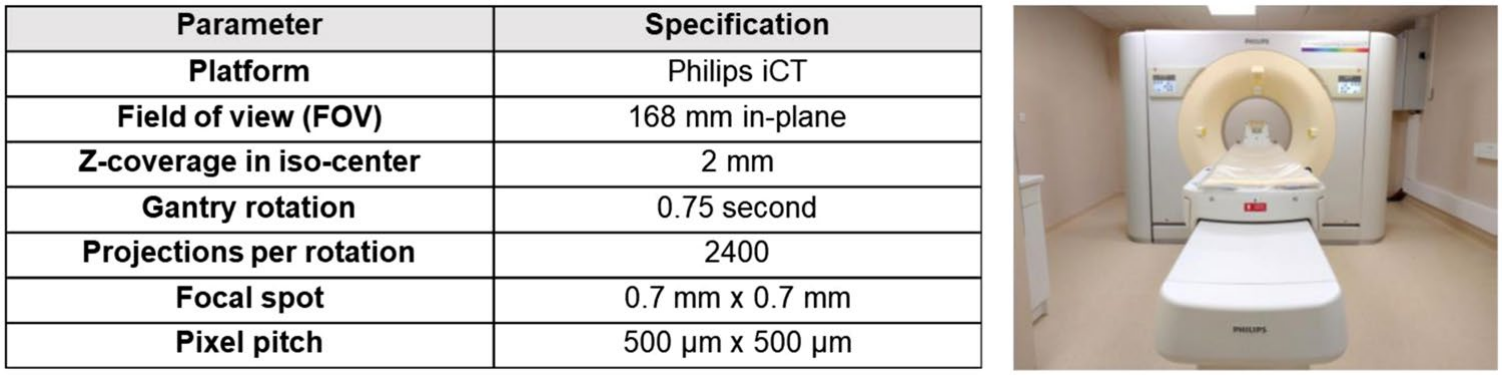
Specifications and photograph of the prototype photon-counting CT scanner used in this study.

A prototype spectral photon-counting CT scanner (Philips Healthcare, Haifa, Israel) modified from a clinical CT base, located at the University of Lyon, was used to acquire phantom images. As previously described^6^, the scanner has field-of-view (FOV) of 168 mm in-plane, z-coverage of 2 mm, gantry rotation time of 0.75 seconds (2400 projection per rotation), focal spot of 0.7 × 0.7 mm, isotropic pixel size of 250 µm, and in-plane resolutions of 11.4 lp/com (line pair per cm) at 50% modulation transfer function (MTF) and 22.4 lp/cm at 10% MTF^23^. The scanner is equipped with photon-counting detectors made of high band gap semiconductor cadmium zinc telluride with a pixel pitch of 500 × 500 µm (Fig. 2)^48^.

The energy-sensitive detectors have 5 configurable energy thresholds. The phantoms were scanned using 5 energy bins whose thresholds were set specifically for each element in this study to allow K-edge imaging (e.g. 30, 51, 78, 83 and 98 keV for gold). For each element, two energy bin thresholds were set just below and above its K-edge for optimal signal-to-noise ratio and accuracy in quantitative information of the element, as described by Roessl *et al*.^49,50^. Five axial scans were performed using a conventional x-ray tube with a voltage of 120 kVp and current of 100 mA. A Philips Ingenuity CT (Philips Healthcare, Cleveland, US), a conventional clinical CT located at the University of Lyon, was used to acquire phantom images. The images were acquired using FOV of 500 mm, 160 mm reconstruction diameter, slice thickness of 1 mm, pixel spacing of 0.3125 mm and gantry rotation time of 0.4 seconds in full 360° scans. The same scan parameters (i.e. tube voltage of 120 kVp and current of 100 mA) were used to acquire the phantom images with both scanners, which are of the same geometry.

### Phantom imaging

**Figure 3:**
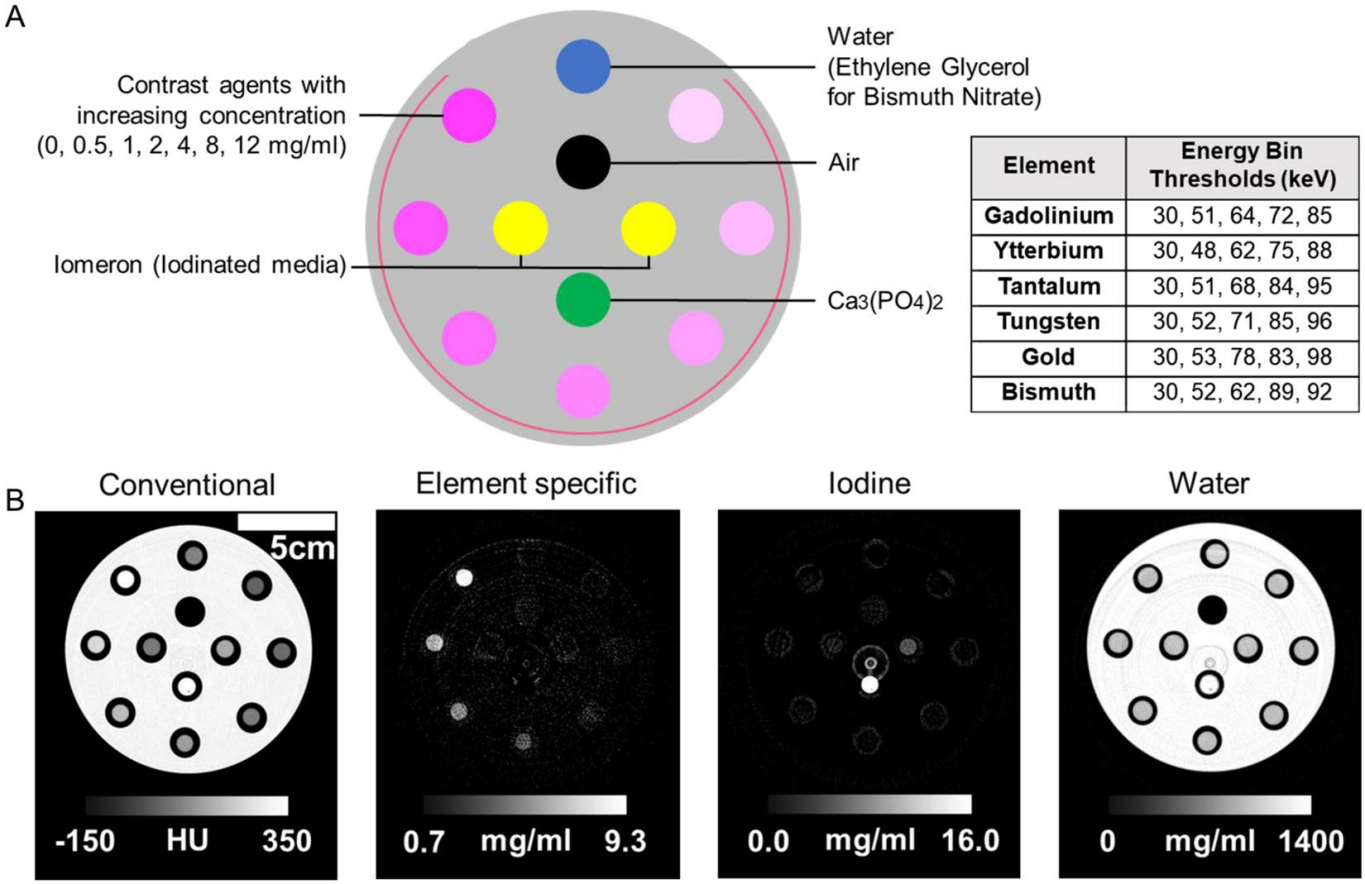
(**A**) Schematic depiction of sample tube locations on the phantom and the energy bin thresholds used for each element. (**B**) Four images generated from SPCCT imaging of gadolinium. Conventional equivalent CT image, element-specific K-edge image of gadolinium, iodine image and water image (left to right).

A polyoxymethylene cylindrical phantom with 13 cm diameter and twelve holes of 1.5 cm in diameter was used as the phantom (Fig. 3A). Seven different concentrations of each element solution (i.e. 0.5, 1, 2, 4, 6, 8, and 12 mg/ml) and the solvent containing no element (ethylene glycol for bismuth nitrate and deionized water for gadolinium acetate, ytterbium chloride, tungsten chloride and gold chloride) were placed in the outer eight holes. Ethylene glycol was used to dissolve bismuth nitrate since it is poorly soluble in water^51^. Two vials containing either 2 mg/ml or 5 mg/ml of iodinated media (Iomeron), one vial containing a bone simulant (calcium phosphate mixed with water) and one empty vial (for air measurement) were placed in the inner four holes. All solutions were prepared in 1.5 ml polypropylene centrifuge tubes (Eppendorf, Hauppauge, NY).

### Material decomposition and image reconstruction

Each slice was reconstructed into conventional CT equivalent images, element specific images, water images, and iodine images (see Fig. 3B for example). Using the energy-dependent information of the transmitted photons in all five energy bins, ‘conventional’ CT images were synthesized, with data displayed in Hounsfield units. Specifically, these images are generated by best fit of the measured count rates in the five energy bins to calibration data based on the phantom with various path lengths. A maximum-likelihood based material decomposition based on literature data of the attenuations of the target materials was applied to derive three material sinograms – element-specific, iodine and water sinograms from the distribution of photon counts across the five energy bins. The material sinograms were then reconstructed separately on an 0.25 × 0.25 × 0.25 mm³ voxel grid using a filtered back-projection algorithm with a standard filter kernel, resulting in three material specific images of the estimated concentration of each material (mg/ml). No further post-processing apart from removal of ring artifacts and smoothing of the element-specific images (i.e. Gaussian blurring of 2 pixels width) was used.

### Image analysis

Attenuation rate (AR) was defined as attenuation divided by the concentration of the elements in mg/ml. CNR was calculated by subtracting the signal in water from the signal of a sample, then dividing the resulting number by the noise (standard deviation in water). CNR rate (CNRR) was defined as CNR divided by the concentration of the element. Noise from the images for each element were determined using OsiriX v.3.7.1 64-bit software (Pixmeo SARL, Bernex, Switzerland). Analysis was done on 2 mm slices. Circular regions of interest (diameter 6.5 mm, area 33.18 mm^2^) were drawn in the middle of each tube, and the attenuation of element concentration from each ROI was recorded. Conventional CT equivalent images were used to calculate AR and element specific K-edge images were used for CNRR. To measure AR, the attenuation values from conventional CT equivalent images (as attenuation on y axis) were graphed against mass concentration of the elements (mg/ml on x axis), and the slope of the line of best fit was calculated^30^. Similarly, the CNR values from element specific K-edge images were compared with element concentrations to determine the CNRR. The noise level was defined as the standard deviation of pixels in the ROI from 0 mg/ml tube.

### Tantalum oxide nanoparticle (TaONP) synthesis

Sub 5-nm TaONP were synthesized by modifying the microemulsion method described by Oh *et al*.^35^. Microemulsions were prepared by adding 250 uL of aqueous 75 mM NaOH solution to 20 mL of cyclohexane containing 2.7 g of IGEPAL CO-520. 50 uL of 0.3 mmol tantalum(V) ethoxide solution (78.3 mg), as provided by the supplier, was added to this emulsion, and the resulting mixture was incubated for 15 min while stirring. To make TaONP water-soluble, 70 uL of 2-(carbomethoxy)ethyltrimethoxysilane and 200 uL of 3-(trimethoxysilyl)propyl-N,N,N-trimethylammonium chloride in 1 ml of ethanol was added to the nanoparticle solution. After 24 hr, a white sediment had formed at the bottom of the flask. The colorless supernatant was removed via careful aspiration, and the white sediment was dissolved in 5 mL of 5 M ammonium hydroxide solution and allowed to stir for 30 min. 25 ml of ultrapure water was added, and the resulting solution was centrifuged at 2500 g for 15 min that resulted in separation of the aqueous phase from remaining cyclohexane and aggregates, which formed a pellet. The clear aqueous solution was collected by careful pipetting, leaving behind the aggregates and cyclohexane. The resulting nanoparticle solution was further purified and concentrated by centrifugation and washing in deionized water three times at 2610 g for 30 min using 10 kDa MWCO centrifugation tubes (GE Healthcare Life Sciences, Marlborough, MA). After each centrifugation, approximately 1 mL of the solution was left at the top compartment of the column, to which 20 mL of deionized water was added for the subsequent centrifugation. Each batch of solution was then redispersed in 4 ml of PBS, centrifuged at 5000 g for 10 minutes in 1.5 mL centrifuge vials, and was filtered through 0.22 μm syringe filter. The final nanoparticle solution was stable in both deionized water and PBS.

### Nanoparticle characterization

Transmission electron microscopy (TEM) was used to evaluate the morphology and core size of TaONP. The microscope used was a JEOL 1010 (JEOL USA Inc., Peabody, MA). ImageJ (NIH) was used to determine the average diameter of nanoparticle cores from the TEM images. The hydrodynamic diameter and ζ-potential of TaONP were determined using a Nano ZS-90 Zetasizer (Malvern Instruments, Worcestershire, UK). The concentration of TaONP was determined through ICP-OES. 5–10 μL samples of nanoparticle solution were dissolved in 1 mL of aqua regia (one part nitric acid to three parts hydrochloric acid) for 1 hr before adding ultrapure water to make the final volume to 5 mL. The resulting solutions were analyzed with a Spectro-Genesis ICP (Spectro Analytical Instruments GmbH, Kleve, Germany) for tantalum concentration.

### *In vitro* experiments

The LIVE/DEAD assay (Life Technologies, Frederick, MD) was used to study the effect of TaONP on the viability of Renca (epithelial kidney cells), SVEC4-10EHR1 (endothelial cells), and HepG2 (hepatocytes). The cells were cultured in 20 mm glass bottom dishes at a seeding density of 1.0 × 10^5^ per dish for 24 hr at 37 °C and 5% CO_2_. The cell growth medium recommended by ATCC for each cell line was used. Next, the media was removed and replaced with fresh media containing various concentrations (0.025 mg/ml to 1 mg Ta/ml) of TaONP. After 8 hr incubation with TaONP, cells were washed twice with DPBS and treated with 400 μL of LIVE-DEAD cocktail (2 μL of ethidium homodimer-1, 1 μL calcein AM, and 3 μL of 3.2 mM Hoechst 33342 in 2 mL DPBS) for 20 min. The cells were then imaged with a Nikon Eclipse Ti-U fluorescence microscope with filters for DAPI, FITC, and Texas Red to visualize cell nuclei, living cells and dead cells, respectively. Images from four different fields of view in each dish were acquired. The images were analyzed by a custom MATLAB code to count the number of cells. The cell viability percent was calculated by dividing the live cell count by total number of cells. These experiments were repeated three times for each cell line.

### Statistical analysis

The LINEST function of Excel was used to determine the slope (*m*_*χ*_) and standard error of fit (*e*_*χ*_) of both AR and CNRR. Following the calculation of *m*_*χ*_ and *e*_*χ*_, the following equation was used to calculate t-values:

$${t}_{1-2}=({m}_{1}-{m}_{2})/\sqrt{({e}_{1}^{2}+{e}_{2}^{2})}$$

The t-values were then compared with the 5% reference t-value at degrees of freedom of 12 (7 concentration points from 1st element (n_1_) + 7 concentration points from 2nd element (n_2_) − 2), which is 2.18. Comparisons were made between every possible pair of the six elements used in this study. Linear regression and Bland-Altman analysis were performed to assess correlation between AR of CT and SPCCT. Two sample T-test was used for *in vitro* experiments to compare the cell viability in each treatment group of various TaONP concentrations to the cell viability in the control group.

## Results

### Phantom imaging

Example conventional CT equivalent, element specific, iodine, and water images generated from scanning the phantom are displayed in Fig. 3B. In the conventional CT equivalent image, an increase in attenuation can be observed as element concentration increases. Element specific images demonstrated that each K-edge element could be successfully differentiated from other materials in the field of view, including calcium phosphate, iodine, and phantom body.

### Attenuation rate

**Figure 4:**
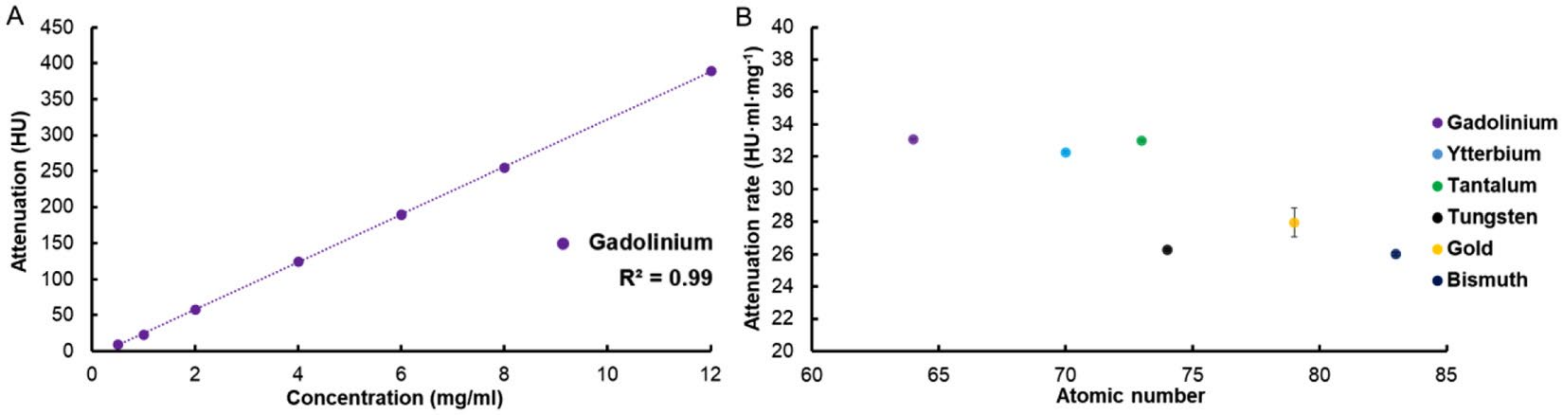
(**A**) Attenuation of gadolinium at a range of concentrations. (**B**) The attenuation rates of the different elements studied. Error bars represent the standard error of the regression. For data points where the error bars are not visible, this is because the error value is very low and is obscured by the data point.

There was an excellent linear correlation between the attenuation observed in conventional CT equivalent images and element concentration used in this study, as is typically the case^6,18,30^. The data for gadolinium is displayed in Fig. 4A as an example. All six elements had an R^2^ value very close to unity (Table S1). We found that gadolinium had the highest value of AR, followed by tantalum, ytterbium, gold, tungsten and bismuth (Fig. 4B). We also observed that there was a noticeable decrease in AR between atomic number 73 (tantalum) and 74 (tungsten). The differences between most of the pairs of elements were statistically significant, except for gadolinium and tantalum, tungsten and bismuth, tungsten and gold, and gold and bismuth (Table S2). It seems that within the section of the periodic table studied and with this scanner, high Z-elements whose atomic number is equal to or less than 73 generate about 20% more attenuation compared to elements whose atomic number is higher than 73, on average.

**Figure 5:**
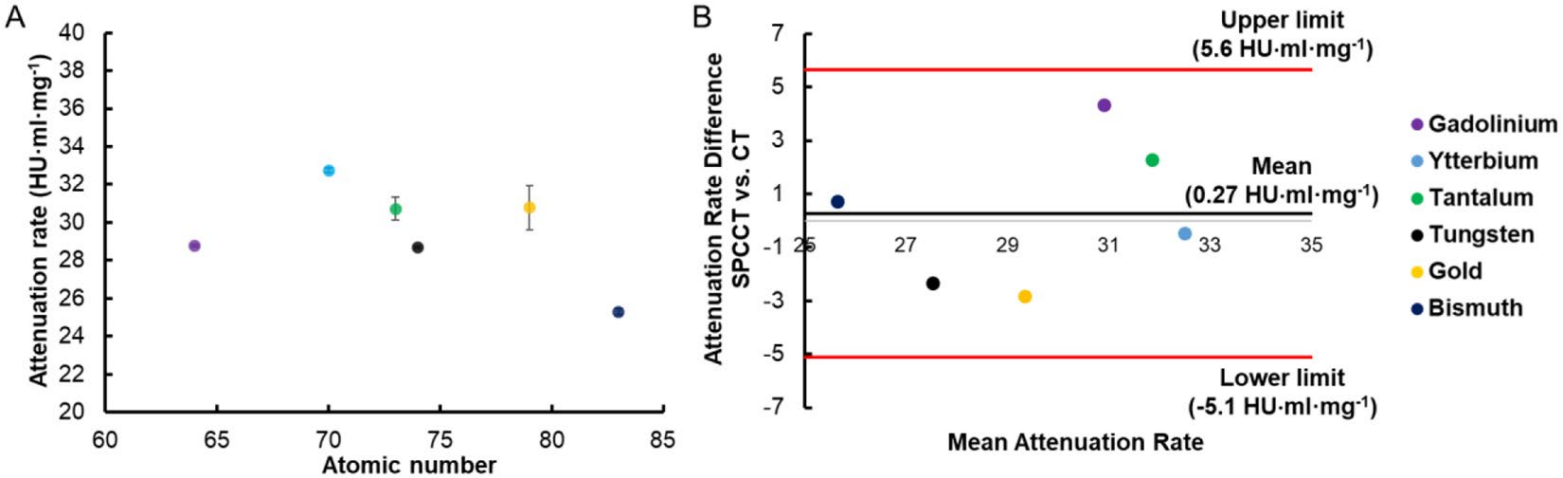
(**A**) The attenuation rates of the different elements studied in conventional CT. (**B**) Bland-Altman analysis of attenuation rate from CT and SPCCT systems. The same key for the elements is used in both A and B.

To examine whether this observed trend in conventional CT equivalent images reconstructed from a SPCCT system was also present in data from EID-based CT images, the phantom was also imaged with a conventional CT scanner with the same tube current and voltage. As can be seen in Fig. 5A, the AR values of the elements from conventional CT images were quite similar to that of the elements from conventional CT equivalent images, ranging from 25.2 to 33.0 HU·ml·mg^−1^ (as opposed to 26.0 to 33.1 HU·ml·mg^−1^ in CT equivalent images from SPCCT). The differences in AR between the elements were less noticeable in conventional CT images, and the decrease in AR between tantalum and tungsten was less apparent. In fact, there was no statistical differences between gold and four elements (gadolinium, ytterbium, tantalum, tungsten), tantalum and two elements (gadolinium and ytterbium), and tungsten and two elements (gadolinium and tantalum) (Table S3).

Bland-Altman analysis was also used to demonstrate the agreement of two AR values between SPCCT and CT. As seen in Fig. 5B, the mean of differences, as known as bias, was 0.27 HU·ml·mg^−1^ with limits of agreement at −5.1 HU·ml·mg^−1^ and 5.6 HU·ml·mg^−1^. The differences in values for all elements in the study were below the limit of agreement and were distributed in a small range near the bias with the highest difference observed in gadolinium (difference of 4.3 HU·ml·mg^−1^). This shows that AR values derived from SPCCT conventional CT equivalent images are comparable to those from conventional CT images.

### Contrast-to-noise ratio rate and noise level

**Figure 6:**
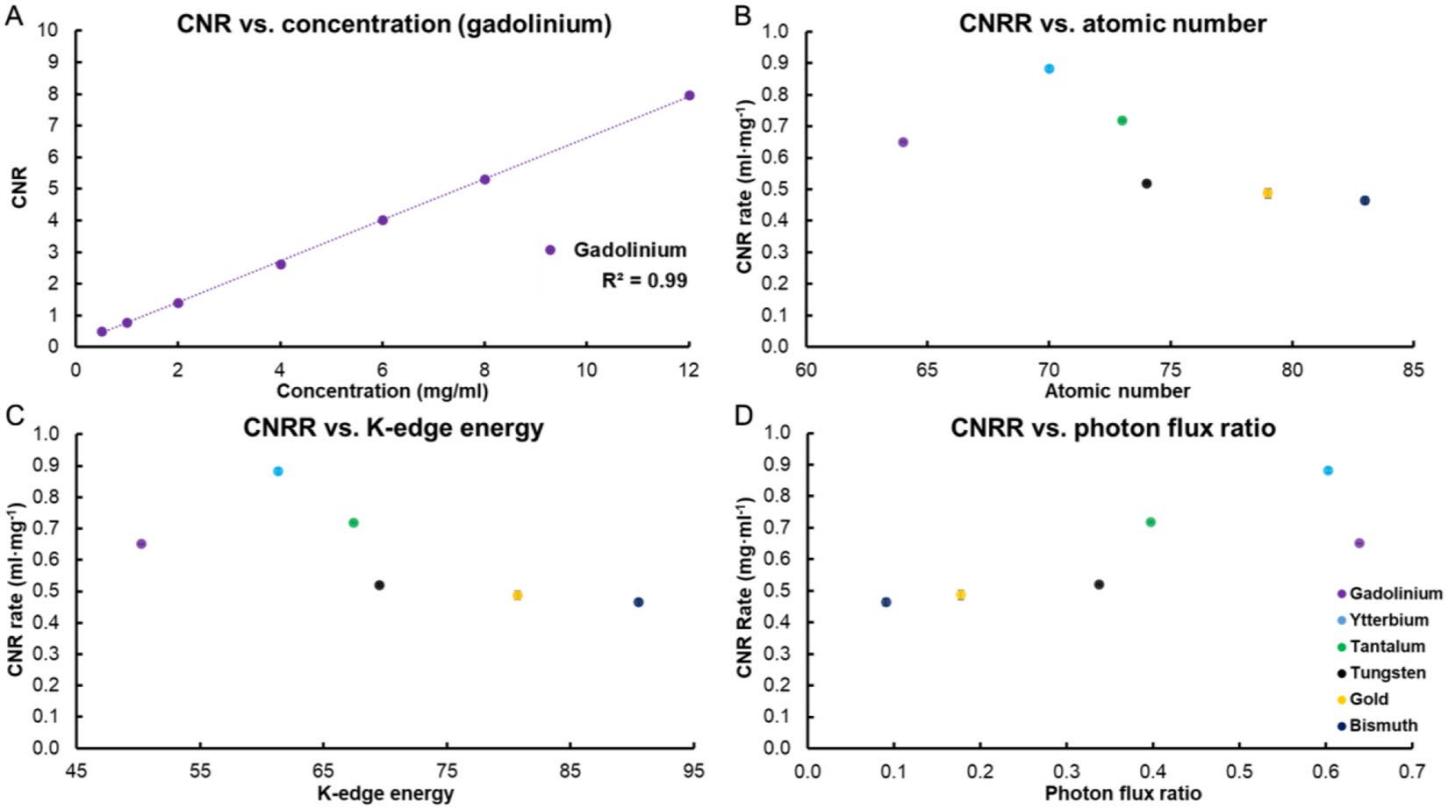
(**A**) CNR of gadolinium at a range of concentrations. (**B**) The CNRR of the different elements studied. (**C**) The CNRR of the different elements compared with their K-edge energies. (**D**) The CNRR of the elements compared to the ratio of the number of photons above and below their K-edge energies (based on the photon spectrum in the air shown in Fig. 1A). In B-D the same key is used. CNR and CNRR were calculated from the element specific images.

As can be seen in Fig. 6A, the CNR values from element-specific images were linearly correlated with element concentration. Similar to AR, CNRR of all six elements had an R^2^ value very close to unity (Table S4), demonstrating the capability of this SPCCT system to accurately quantify the concentrations of the elements, similar to results found for gold previously^6,18^.

**Figure 7:**
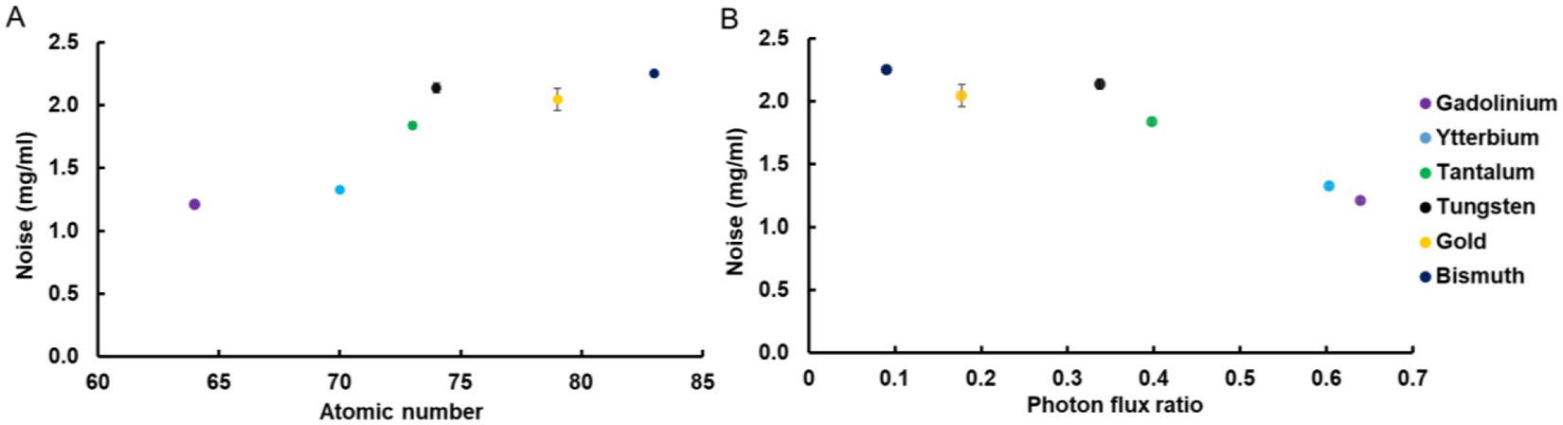
(**A**) Noise levels of the element specific images. (**B**) Noise level of the element specific images of the elements compared to the ratio of the number of photons above and below their K-edge energy.

CNRR values were the highest for ytterbium, followed by tantalum, gadolinium, tungsten, gold and bismuth (Fig. 6B). There were no statistically significant differences in CNRR values between gold and bismuth as well as gold and tungsten, but the CNRR values were found to be statistically significantly different between all other possible pairs of elements (Table S5). The differences were also substantial, with the CNRR for ytterbium being 62% and 58% higher than those for bismuth and gold, respectively. Similar to AR, CNRR was lower for elements whose atomic number was 74 or above, compared with elements 73 or lower. This created two groups of elements, one that has high CNRR (gadolinium, ytterbium, tantalum) and the other low CNRR (tungsten, gold, bismuth). In an effort to understand the variation in CNRR, we examined its relationships with other parameters, such as the K-edge energy of the elements and the ratio of number of photons above and below the K-edge energy. As can be seen in Fig. 6C, the elements whose K-edge energies are at 67.4 keV (tantalum) or below have higher CNRR than the elements whose K-edge energies are at 69.5 keV (tungsten) or above. Having sufficient number of photons below and above the K-edge energy is considered to be a factor in ideal reconstruction of element specific images. Thus, we also analyzed CNRR in terms of the photon flux ratio above and below the K-edge energies. We observed that higher photon flux ratios closer to one resulted in higher CNRR values (Fig. 6D). Last, we examined the noise in the element specific images (Fig. 7). The noise was the main determinant of CNRR in SPCCT imaging, since low noise was observed for the elements whose atomic number is less than or equal to 73, while higher noise was observed in elements whose atomic number is higher than 73 (Fig. 7A). We also observed that noise level generally decreases with increasing photon flux ratio above and below the K-edge energies (Fig. 7B).

### TaONP synthesis and *in vitro* biocompatibility

**Figure 8:**
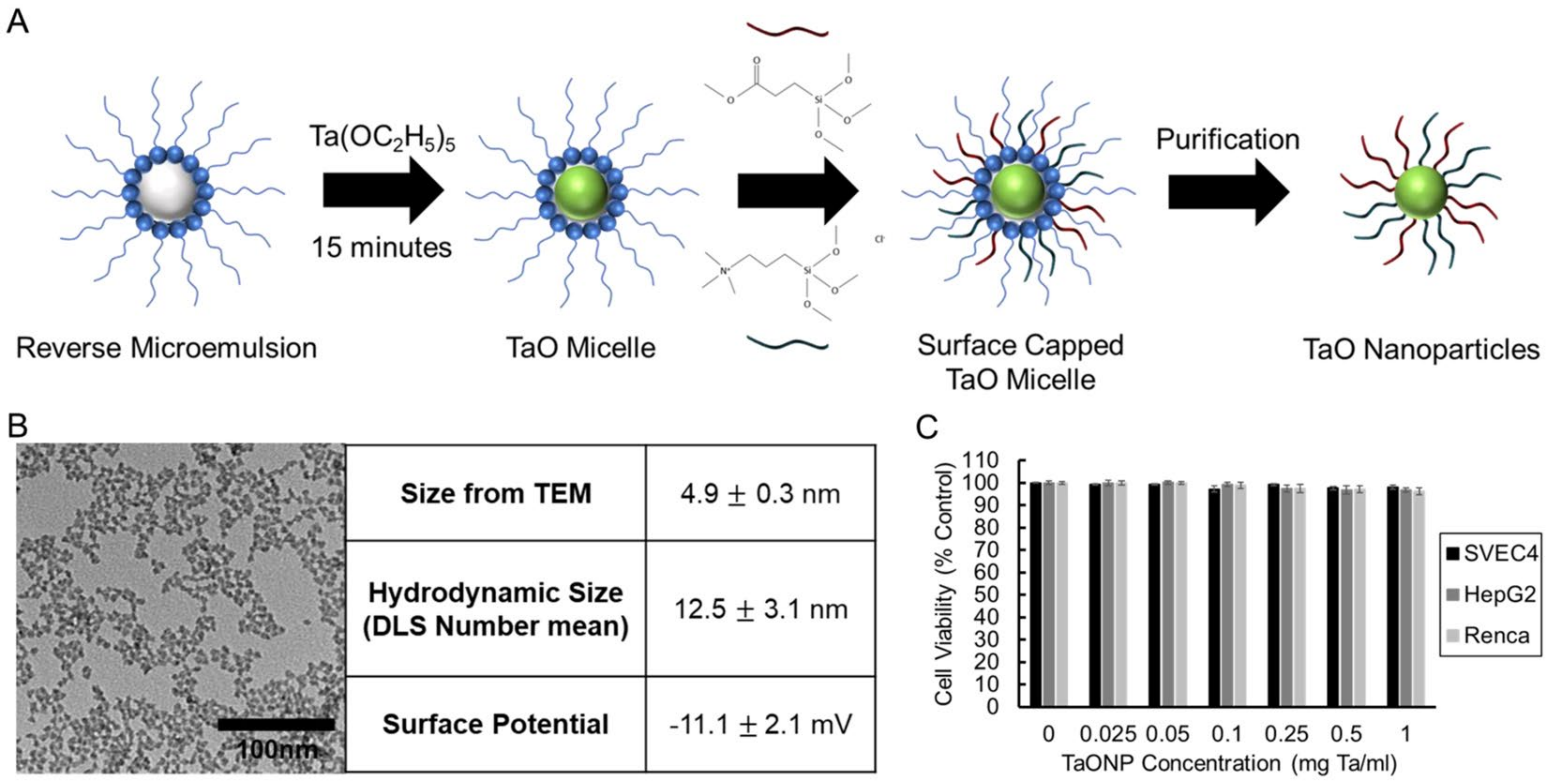
(**A**) Schematic depiction of the TaONP synthesis process. (**B**) TEM of TaONP, size and surface potential of TaONP measured from TEM and DLS. (**C**) Effect of TaONP on cell viability after 8 hr of incubation.

As demonstrated in the phantom imaging, tantalum showed one of the highest AR values along with gadolinium, and the second highest CNRR value, after that of ytterbium. Moreover, its high elemental density (d = 16.4 g/cm^3^), affordability (approximately 13-fold cheaper than gold and 4-fold cheaper than ytterbium), and, most importantly, K-edge energy that can lead to material differentiation from calcified structures in SPCCT, makes tantalum an appealing element for further development into a SPCCT contrast agent. To test the potential use of tantalum-based contrast agents in SPCCT imaging, water-soluble, sub-5 nm TaONP were synthesized. TaONP were synthesized using a microemulsion method, and given a zwitterionic small molecule coating to provide solubility and stability in biological media (Fig. 8A)^52^. The size and surface potential of TaONP were characterized by TEM and DLS (Fig. 8B). The mean core diameter measured from TEM images was 4.9 nm. The mean hydrodynamic diameter of 12.5 nm from DLS measurements was larger as expected, due to the molecules attached to the nanoparticle surface. The surface potential of TaONP was found to be slightly negative (−11.1 ± 2.1 mV).

To study the safety of TaONP, they were incubated with cell types that are expected to have the high exposure upon injection and blood circulation, i.e. hepatocytes, endothelial cells and epithelial kidney cells (Fig. 8C). There was no statistically significant effect of the TaONP incubations for any cell type or concentration, indicating their safety.

### TaONP in SPCCT phantom imaging

**Figure 9:**
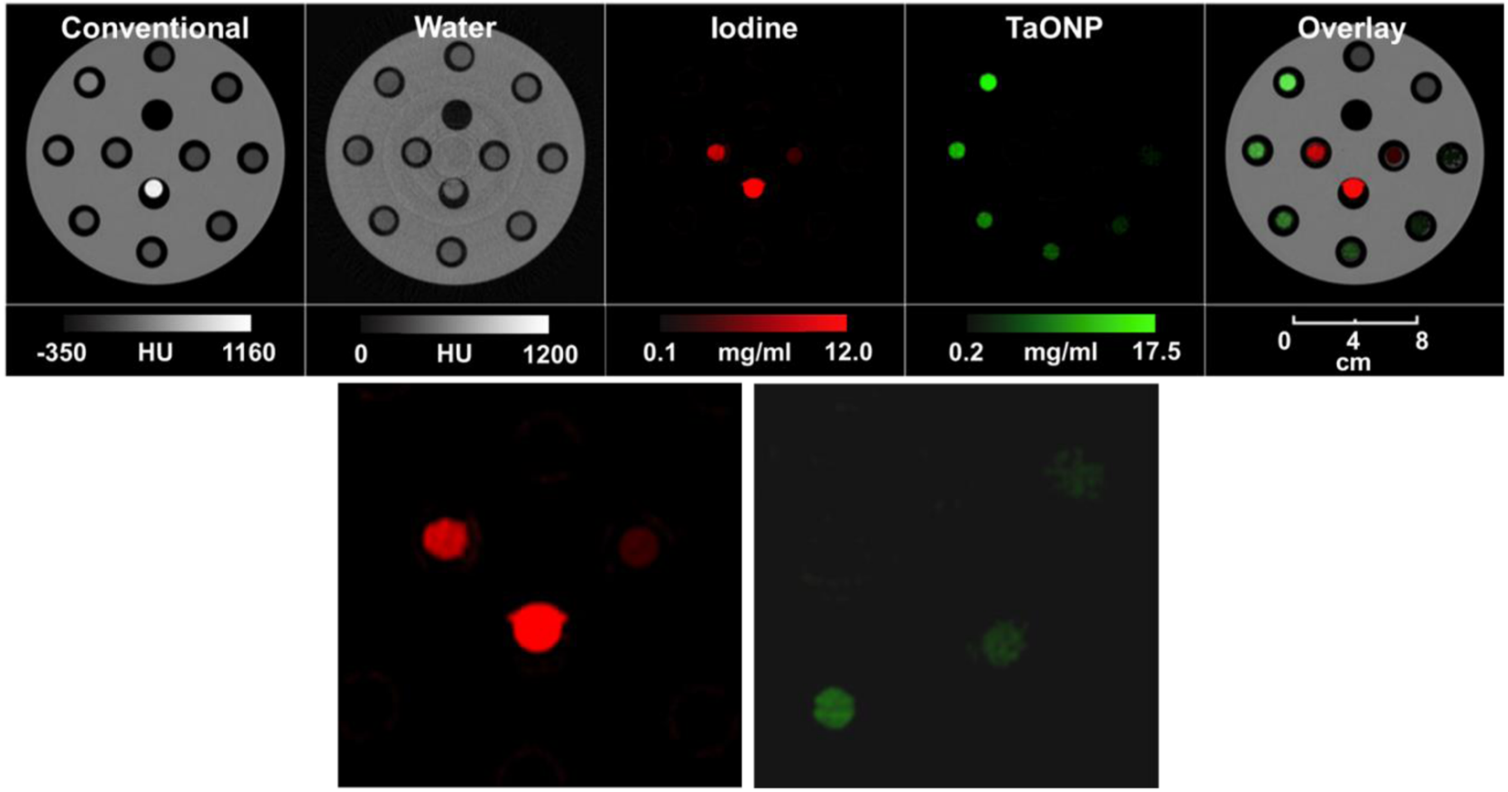
SPCCT images of the phantom showing differentiation of TaONP ranging from 0 to 12 mg Ta/ml from an iodine contrast agent (2 and 5 mg/ml). Two images on the bottom row are the enlarged iodine and TaONP images centered at tubes with lower element concentrations.

To study the capabilities of the SPCCT system in differentiating TaONP from other elements (i.e. iodine) and the detection limit of TaONP, phantom images of TaONP were acquired. As seen in Fig. 9, the scanner could accurately determine the location of TaONP in the field of view. Iodine and calcium phosphate were clearly distinguished from TaONP. TaONP-specific images indicate that concentrations to 1 mg/ml of TaONP can be detected in the SPCCT scanner, which is comparable to the gold detection limit of 1 mg/ml in conventional CT scanner^30^.

## Discussion

We have demonstrated that gadolinium, ytterbium and tantalum provide higher CT signal generation and better element-specific image CNR in the current prototype SPCCT system used in this study, over tungsten, gold and bismuth. These results suggest that the elements in the range of atomic numbers between 64 to 73 can be suitable candidates for future development into high-performing contrast agents for SPCCT imaging. However, we observed a significant decrease in attenuation between atomic number 73 and 74 (tungsten). This is possibly due to the fact that tungsten is the material of the x-ray tube anode, which causes K-edge absorption within the tube at the K-edge energy of tungsten. In particular, gadolinium, ytterbium, tantalum and likely elements close to them in atomic number (e.g. europium, terbium, hafnium) can potentially generate the most contrast in SPCCT. These outcomes can be explained by the fact that gadolinium, ytterbium and tantalum have their K-edges at energies where there are high numbers of photons both above and below that energy in a 120 kVp beam, resulting in better data statistics for more accurate material decomposition and hence lower image noise. These findings in our study were demonstrated at a scanning condition of 120 kVp peak tube voltage. It is important to note that gold and bismuth – two elements that generated less contrast in this study – will likely produce stronger contrast at 140 kVp since higher number of photons will be generated near (especially above) their K-edge energies, compared to when 120 kVp is used. Several studies have examined nanoparticle development and small animal imaging with ytterbium^53–55^ and tantalum^34,35,56^, indicating the potential of these two elements for further development for SPCCT contrast agents. However, more research is needed to evaluate nanoparticle behaviors, such as long-term *in vivo* cytotoxicity, biodistribution and optimal dosage. Ease of size control and surface coating make tantalum a more desirable element for nanoparticle-based contrast agent development than ytterbium.

For phantom images provided in Fig. 3, iodine and water images were constructed in a similar manner as dual energy CT images in which all materials are mapped; therefore, materials other than water and iodine are also seen in these images. Since the element samples were dissolved in water or a water-like solvent (i.e. ethylene glycol in the case of bismuth), there is some signal from these samples in the water image (Fig. 3B). Similarly, the plastic of the phantom also appears on the water images due to the fact that it is composed of elements similar to the atomic number to hydrogen and oxygen. This also explains the slightly larger appearance of the samples in water images since the plastic tubes containing the samples also appear. In all cases, signal from calcium phosphate is seen in iodine images and in water images with higher attenuation than other elements since the material decomposition algorithm classifies the attenuation pattern of calcium to be in between that of iodine and water. This is in agreement with observations made in previous studies using SPCCT^6,18^.

Optimization of energy windows is an important issue for K-edge imaging since the energy thresholds of each energy bin greatly affect the material decomposition as well as CNR of the materials in SPCCT imaging. The selection of energy bins determines the spectral separation and the count rate of each energy bin. Thus, they have to be optimized for given set of materials. For K-edge of an element to be detected, the energy bins must properly capture the K-edge. This is typically done by assigning the energy thresholds of a bin just below and above the K-edge energy. Before our study, energy thresholds for all five energy bins were optimized for each element used in this study, based on the methods described by Roessl *et al*. to minimize the noise in material decomposition and optimize the signal-to-noise ratio in material-specific images of each element^49,50^. For example, energy thresholds of 30, 51, 78, 83, and 98 keV were used for gold whose K-edge energy is 80.7 keV.

Different types of material decomposition algorithms can lead to different degrees of signal-to-noise ratio degradation and variances in noise. In this study, we performed projection space-based material decomposition. However, decomposition can be performed on reconstructed images, which can yield different noise levels^57^. Recently, one-step inversion algorithms called statistical iterative reconstruction techniques that can improve image quality has been investigated by Mechlem *et al*.^58^. The continued improvement in material decomposition techniques can lead to decreased noise levels in material-specific images of all six elements in this study.

The proof-of-concept experiments with tantalum-based nanoparticles confirmed that, as others have found, biocompatible nanoparticles can be made with this element^35,59^. In addition, we showed for the first time that these nanoparticles can be successfully differentiated from calcium phosphate and iodine with SPCCT. TaONP were distinguished from the other elements, and their detection limit was comparable to that of gold in conventional CT systems (close to 1 mg/ml). Besides the successful differentiation and comparable detection limit, the biodistribution as well as pharmacokinetics of these nanoparticle-based contrast agents can be controlled by adjusting the size and the surface chemistry to lessen renal damage and optimize the imaging window, which can provide further advantages over iodinated contrast agents^60^. By using improved image reconstruction methods (i.e. iterative reconstruction), reconstruction kernels and post-image processing (i.e. removal of ring artifacts and Gaussian blurring) that can lower the noise, it is expected that the detection limit of TaONP as well as other heavy metal elements can be lowered^61^.

Gadolinium, ytterbium and tantalum can be particularly valuable elements for further developments into SPCCT contrast agents, considering toxicity, cost, availability, previous uses in nanoparticle synthesis and developmental contrast agents. We also demonstrated that these elements could also work well in conventional CT scanners, which can provide additional utility of the contrast agents made of these elements.

Gadolinium-based contrast agents are commercially available and are widely used for clinical uses in MR imaging^62^. However, due to lower sensitivity in CT scanners, higher doses of gadolinium will likely be needed for SPCCT imaging, which may result in toxicity issues. Indeed, there is considerable concern over brain retention of gadolinium-based agents^63^. A clinical study is currently evaluating feasibility of using low-dose of gadolinium-based contrast agents for CT imaging^64^. A recent study also demonstrates the feasibility of acquiring images of diagnostic quality with gadolinium-based contrast agents at clinical MRI routine doses of 0.2 mmol/kg in human patients via spectral CT imaging^65^. If the sensitivity of SPCCT technology – scanners and reconstruction algorithms - is improved, the required doses of gadolinium-based contrast agents for *in vivo* imaging may be further reduced^61^. Nevertheless, further work is needed to fully understand the potential use of the currently available gadolinium-based agents for SPCCT imaging.

Although contrast generation is a valuable assessment of an element’s potential SPCCT imaging performance, it is one of several factors that need to be considered for CT imaging applications. For instance, in the case of cell tracking there is a need for high contrast agent density, to allow cells to take up high payloads, as well as very high biocompatibility and stability. To date gold nanoparticles have almost exclusively been used for cell tracking with CT^1^ since they meet all of the above criteria, and they therefore may remain the platform of choice for cell tracking with SPCCT, despite the sub-optimal CNR. Inferior CNR of gold found in this study is likely due to the fact that we were using tube voltage of 120 kVp. As mentioned earlier in discussion, gold is likely to provide better contrast at 140 kVp^30^. Furthermore, gold nanoparticles have been very frequently used as experimental CT contrast agents in general, due in part to the ability to synthesize them in a wide variety of sizes and shapes, as well as the ability to conjugate them with targeting ligands, each of which is easier than for elements such as tantalum^66–68^. We may therefore continue to see reports of gold nanoparticle SPCCT agents.

Our study provides insight into potential elements that can be developed as contrast agents for SPCCT imaging, but it has limitations. Similar studies will need to be performed in other SPCCT systems to further validate the potential elements for SPCCT contrast agents as variations in system specifications may result in different trends in CT attenuation and CNR. Moreover, the SPCCT system used in this study is a prototype of a clinical scanner under development. The final clinical scanner may have a different x-ray source, detector architecture and may use different scanning conditions, which could result in some differences with the values reported herein. When used with a larger FOV, larger subjects, such as human patients, may affect CNR production via the beam hardening effect, causing fewer photons below the K-edge energies of these elements to reach the detector. In this case, elements with higher atomic numbers will therefore produce higher CNR than for smaller subjects. This might lead to, for example, the contrast generation of tantalum being higher than that of ytterbium. All of these limitations of the study can be addressed in the future in larger phantoms or animal models as the technology approaches the clinical stage of the development.

In summary, our study’s results suggest that gadolinium, ytterbium and tantalum have high attenuation and contrast-to-noise ratio in SPCCT, indicating that these elements may be superior candidates for further development into novel contrast agents for SPCCT imaging. Our data also suggests that these elements can potentially be effective as contrast generating media for conventional CT scanners. Lastly, we have shown that tantalum can be developed into nanoparticles that are stable and biocompatible for biological applications. These nanoparticles could be differentiated from water, iodine and calcium phosphate in SPCCT, providing potential applications in the field of cardiovascular and cancer imaging.

## Electronic supplementary material

Supplemental Informtion

## Data Availability

All data generated or analyzed during this study are included in this published article (and its Supplementary Information files).
