# Supplementary material for: Assessment of candidate elements for development of spectral photon-counting CT specific contrast agents
Source: Sci Rep. 2018 Aug 14;8:12119. doi: 10.1038/s41598-018-30570-y (PMC6092324; doi:10.1038/s41598-018-30570-y)
Supplement: Supplementary file 1 — Supplemental Informtion [file 41598_2018_30570_MOESM1_ESM.pdf]

Assessment of candidate elements for development of spectral photon-counting CT specific contrast agents

Johoon Kim,[1,2] Daniel Bar-Ness,[4,5] Salim Si-Mohamed,[4,5] Philippe Coulon,[6] Ira Blevis,[7] Philippe Douek,[4,5] David P. Cormode[1,2,3]*

Departments of Radiology[1], Bioengineering[2], Medicine, Division of Cardiovascular Medicine[3], University of Pennsylvania, 3400 Spruce St, 1 Silverstein, Philadelphia, PA 19104, USA, Tel: 215-615-4656, Fax: 240-368-8096 david.cormode@uphs.upenn.edu

Department of Radiology[4], Hôpital Cardio-Vasculaire et Pneumologique Louis Pradel, Lyon, France

Centre de Recherche en Acquisition et Traitement de l'Image pour la Santé (CREATIS)[5], UMR CNRS 5220, Inserm U1044, University Lyon1 Claude Bernard, Lyon, France

CT Clinical Science[6], Philips, Suresnes, France

Global Advanced Technologies[7], CT, Philips, Haifa, Israel

* Corresponding Author

Element	R^2 value of AR
Gadolinium	0.9999
Ytterbium	0.9999
Tantalum	0.9999
Tungsten	0.9999
Gold	0.9806
Bismuth	0.9996

Supporting Table 1. R^2 value of AR in all six elements in the phantom study.

vs.	Gadolinium	Ytterbium	Tantalum	Tungsten	Gold	Bismuth
Gadolinium		-3.81	-0.55	-37.26	-2.91	-25.94
Ytterbium	3.81		3.62	-28.61	-2.45	-21.55
Tantalum	0.55	-3.62		-40.72	-2.86	-26.71
Tungsten	37.26	28.61	40.72		0.89	-1.35
Gold	2.91	2.45	2.86	-0.89		-1.09
Bismuth	25.94	21.55	26.71	1.35	1.09	

Critical t- value	2.18

Supporting Table 2. T-values of every possible pairs of elements in two sample T-test for AR in conventional CT equivalent images of SPCCT. The pairs that did not have statistically significant differences in AR are highlighted in gray.

vs.	Gadolinium	Ytterbium	Tantalum	Tungsten	Gold	Bismuth
Gadolinium		13.95	1.63	-0.29	0.88	-9.27
Ytterbium	-13.95		-1.63	-13.96	-0.84	-17.03
Tantalum	-1.63	1.63		-1.67	0.02	-4.32
Tungsten	0.29	13.96	1.67		0.90	-9.07
Gold	-0.88	0.84	-0.02	-0.90		-2.35
Bismuth	9.27	17.03	4.32	9.07	2.35	

Critical t- value	2.18

Supporting Table 3. T-values of every possible pairs of elements in two sample T-test for AR in conventional CT images. The pairs that did not have statistically significant differences in AR are highlighted in gray.

Element	R^2 value of CNRR
Gadolinium	0.9997
Ytterbium	0.9977
Tantalum	0.9996
Tungsten	0.9991
Gold	0.9819
Bismuth	0.9879

Supporting Table 4. R^2 value of CNRR in all six elements in the phantom study.

vs.	Gadolinium	Ytterbium	Tantalum	Tungsten	Gold	Bismuth
Gadolinium		11.86	8.21	-14.80	-5.45	-7.88
Ytterbium	-11.86		-8.28	-18.06	-11.29	-14.08
Tantalum	-8.21	8.28		-21.18	-7.66	-10.66
Tungsten	14.80	18.06	21.18		-1.10	-2.33
Gold	5.45	11.29	7.66	1.10		-0.60
Bismuth	7.88	14.08	10.66	2.33	0.60	

Critical t- value	2.18

Supporting Table 5. T-values of every possible pairs of elements in two sample T-test for CNRR in element specific images of SPCCT. The pairs that did not have statistically significant differences in CNRR are highlighted in gray.
